# Anesthetic Management of Inguinal Hernia Surgery Using a Second-Generation Supraglottic Airway in a Patient With Trisomy 18: A Case Report

**DOI:** 10.7759/cureus.45337

**Published:** 2023-09-16

**Authors:** Shintaro Akimaru, Toshiyuki Nakanishi, Tatsuya Hasegawa, Kazuya Sobue

**Affiliations:** 1 Department of Anesthesiology and Intensive Care Medicine, Nagoya City University Graduate School of Medical Sciences, Nagoya, JPN

**Keywords:** trisomy 18 syndrome, pediatric anesthesia, supraglottic airway device, inguinal hernia repair, airway management

## Abstract

Children with trisomy 18 have abnormal airway anatomy, making their airway management challenging. Only a few papers have comprehensively described and discussed the use of supraglottic airway devices in patients with trisomy 18. We present a case of a 20-month-old boy with trisomy 18 who was scheduled for open repair of the right inguinal hernia. He had micrognathia, a short neck, and an atrial septal defect but was in a clinically stable condition. A supraglottic airway device was inserted under general anesthesia. The patient’s respiration was maintained by pressure support ventilation with spontaneous breathing. A right ilioinguinal-iliohypogastric nerve block was performed for perioperative analgesia. The surgery ended without complications. After removing the supraglottic airway device and ensuring proper respiratory parameters, the patient was transferred to the post-anesthesia care unit. In our case, supraglottic airway devices could be effectively used as a primary airway for inguinal hernia repair. The concomitant ilioinguinal-iliohypogastric nerve block was helpful for anesthetic management with spontaneous breathing maintained using pressure support ventilation. A supraglottic airway device may be a potential alternative as a primary airway for superficial surgery in pediatric patients with trisomy 18. For pediatric patients with difficult airways, a second-generation supraglottic airway device with the insertion of a gastric tube to prevent gastric insufflation combining pressure support ventilation and positive end-expiratory pressure may be a beneficial choice for the maintenance of spontaneous breathing.

## Introduction

Trisomy 18 is a common autosomal chromosomal abnormality characterized by malformations throughout the body and occurs in approximately 1/3,600 to 1/10,000 live births [1]. Approximately 50% of newborns with trisomy 18 expire within the first week of life, with only 10% reaching their first birthday [2]. Children with trisomy 18 may require gastrointestinal, airway, or cardiac surgery during infancy. The abnormal airway anatomy, including micrognathia, high-arched palate, and short neck makes mask ventilation, and tracheal intubation challenging [2].

Supraglottic airways (SGAs) play an important role in difficult airway management in both adults and children [3]. Previous studies of children with trisomy 18 have focused on performing tracheal intubation [2,4]. However, only a few have described and discussed the use of SGAs to maintain general anesthesia in patients with trisomy 18 [3,5,6]. Thus, the detailed assessment and management strategy of SGAs in children with trisomy 18 is unclear.

In this report, we describe the successful use of an SGA in an infant with trisomy 18 undergoing inguinal hernia repair. Written informed consent for publication was obtained from the patient’s parents.

## Case presentation

The patient was a 20-month-old boy with a height and weight of 65 cm and 6.4 kg, respectively. During the fetal period, the patient was diagnosed with trisomy 18. The patient was born by cesarean delivery at week 38 of gestation with a low birth weight (1,942 g). At birth, the patient required mechanical ventilation and tracheal intubation for seven days because of respiratory failure. The neonatologist successfully performed tracheal intubation on the third attempt using a direct laryngoscope. The intubation procedure took eight minutes, during which there was one esophageal intubation with a minimum percutaneous oxygen saturation (SpO_2_) of 75%. Although diagnosed with a right inguinal hernia at birth, the patient had been observed without surgical intervention. Owing to concerns of bowel obstruction, open repair of the right inguinal hernia was scheduled at 18 months of age.

**Figure 1:**
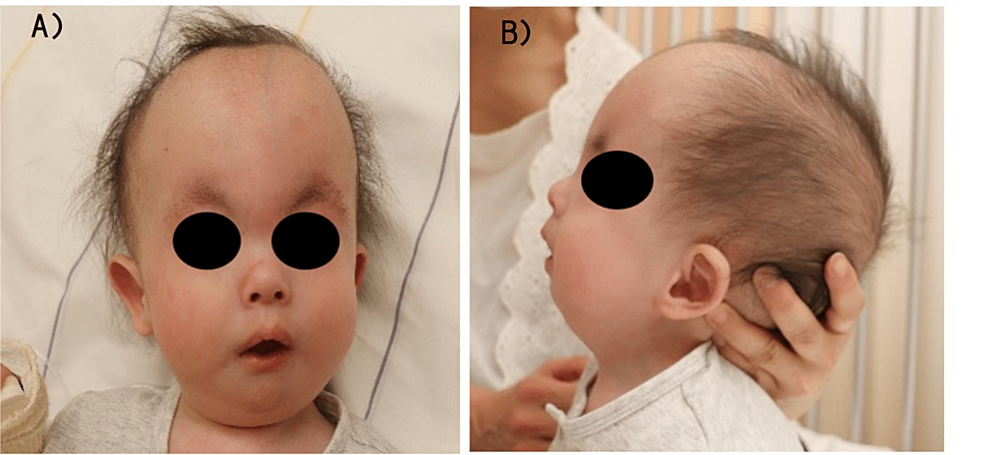
Preoperative frontal (A) and lateral (B) views of the patient Preoperative airway assessment revealed that the patient had normal cervical retroflexion, micrognathia, and a short neck.

One week before surgery, preoperative airway assessment revealed normal cervical retroflexion, micrognathia, and a short neck (Figure 1). The patient could sleep in the supine position without experiencing respiratory distress, such as sleep apnea, necessitating oxygen therapy or continuous positive airway pressure (CPAP). Preoperative transthoracic echocardiography showed an atrial septal defect with a left-to-right shunt but without other structural or mechanical cardiovascular problems, indicating hemodynamic stability. Findings of other preoperative examinations, including blood tests (complete blood counts, coagulation studies, and biochemical examinations), electrocardiography, and chest X-ray imaging, were not significant. The patient was classified as American Society of Anesthesiologists (ASA) physical status III and considered suitable for general anesthesia management.

**Figure 2:**
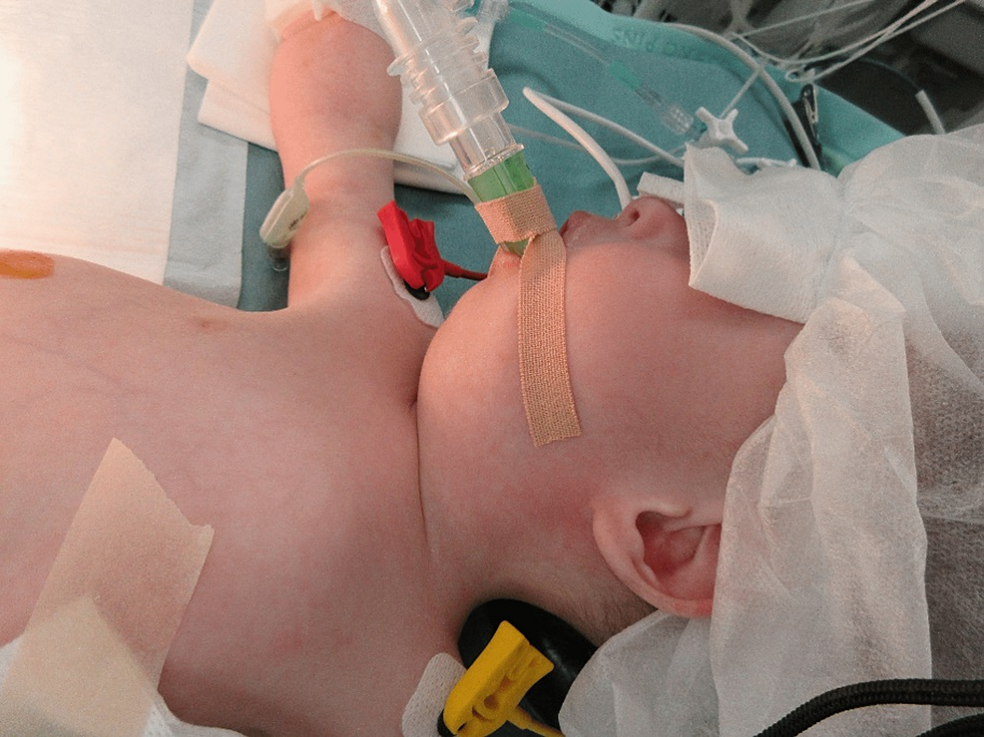
AuraGain inserted in the patient The AuraGain was smoothly inserted and fit well.

**Figure 3:**
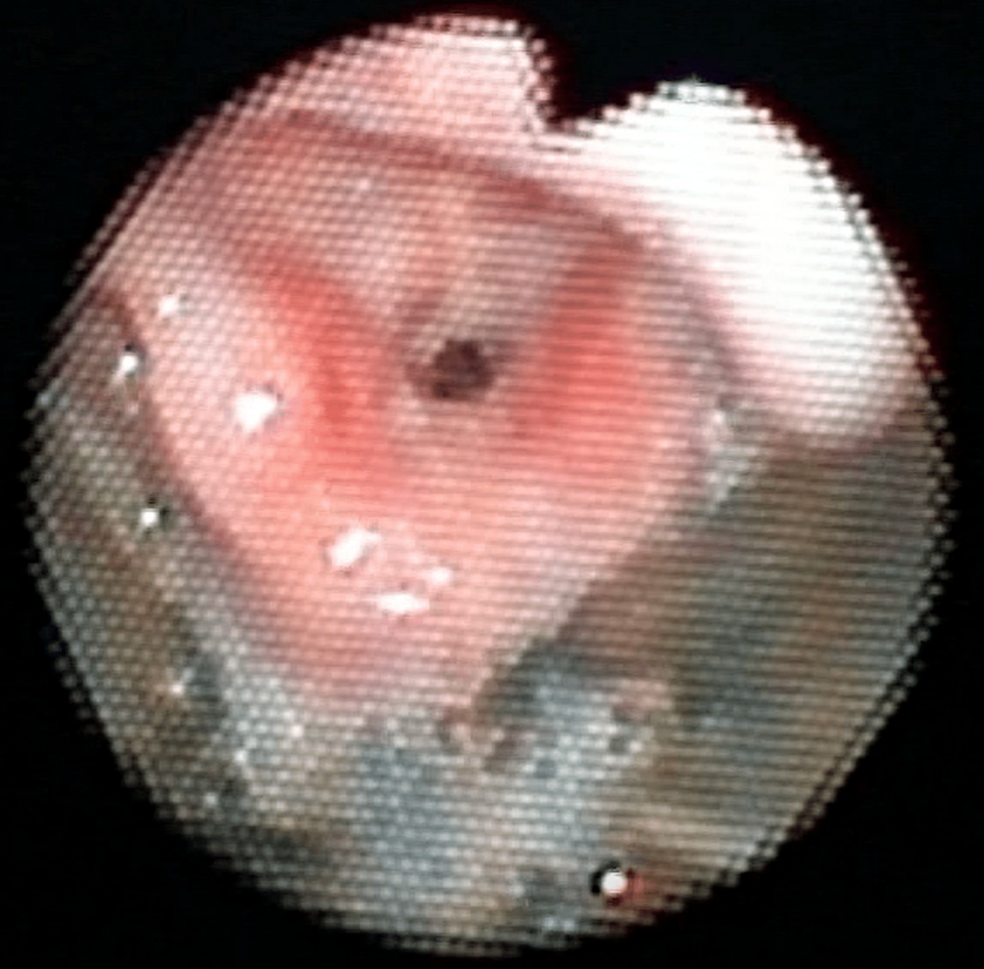
Bronchial fiber view of the patient’s larynx via the AuraGain The epiglottis was lifted, and the glottis could be seen in the middle.

General anesthesia with an SGA was scheduled as the primary airway for intraoperative management. On the day before surgery, a 24-G venous catheter was inserted, and an 8-Fr nasogastric tube was placed to facilitate tracheal intubation and avoid aspiration caused by gastric distention during difficult mask ventilation from our own experience. Standard monitoring instruments, including an oxygen saturation probe, a non-invasive blood pressure monitor, and electrocardiogram probes, were attached. General anesthesia was induced by the administration of 5% sevoflurane in 100% oxygen in conjunction with intravenous atropine (0.016 mg/kg) and fentanyl (0.78 µg/kg) without neuromuscular blocking drugs. Mask ventilation with the one-handed technique was difficult but was facilitated with the two-handed technique. A size 1.5 Ambu®AuraGain™ (Ambu, Ballerup, Denmark) was inserted and well-fitted (Figure 2). Anesthesia was maintained with sevoflurane with a minimum alveolar concentration of 0.8 in 40% oxygen and 10 µg of intravenous fentanyl. Intraoperatively, the patient’s respiratory system was maintained by pressure support ventilation (PSV). A PSV of 12 cmH_2_O, positive end-expiratory pressure (PEEP) of 5 cmH_2_O, trigger flow of 1.0 L/minute, and a rate of 25/minute were used with the Dräger Perseus A500 (Dräger, Lübeck, Germany). The larynx was observed by inserting a 2-mm diameter bronchial fiberscope (BF) through the AuraGain (Figure 3). The optimal positioning of the AuraGain was confirmed by visualizing the vocal cords and posterior epiglottis with the BF [7].

**Figure 4:**
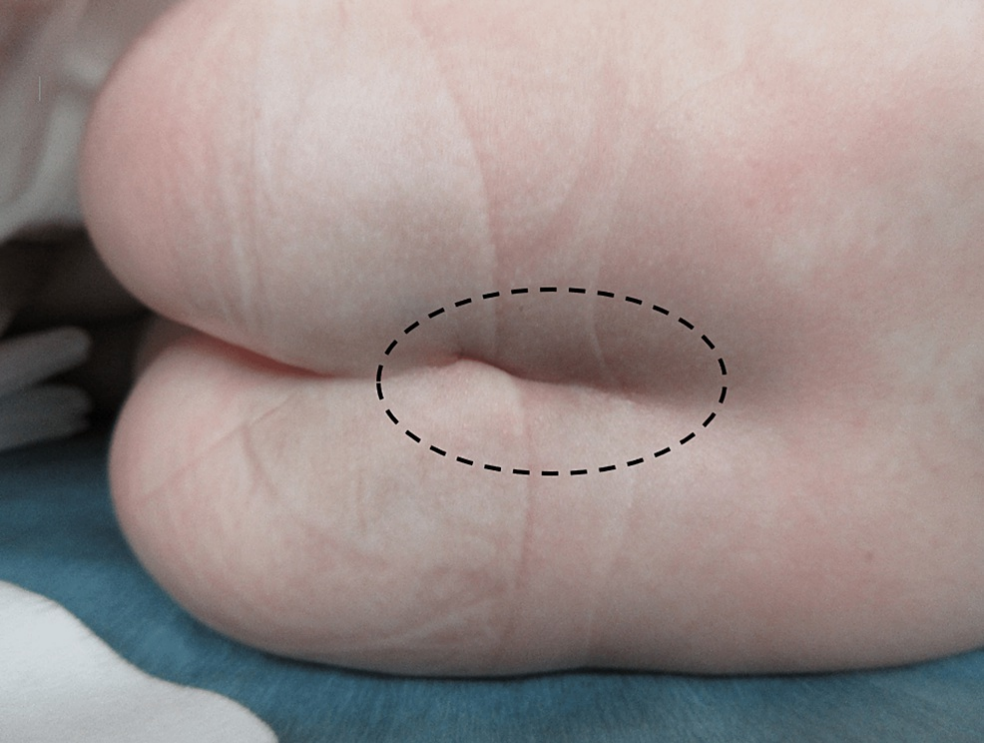
Patient’s back appearance after anesthesia induction The black circle shows the suspected region of a sacrococcygeal dermal sinus.

Initially, perioperative analgesia with caudal epidural analgesia was planned. However, this original plan was abandoned because of a suspected sacrococcygeal dermal sinus found in the patient’s buttocks (Figure 4). Thus, a right ilioinguinal-iliohypogastric nerve block (IINB) with 8 mg of 0.2% levobupivacaine was performed for intraoperative and postoperative analgesia.

Throughout the procedure, the patient’s circulatory and respiratory status remained stable, with SpO_2_ maintained at approximately 99%, a tidal volume of 65 mL, and a respiratory rate of 25 breaths per minute. The procedure lasted 44 min without complications.

After the patient was awakened, the AuraGain was removed. After ensuring proper airway and breathing, the patient was transferred to the post-anesthesia care unit (PACU). The patient's behavioral observational pain scale was zero, and no additional analgesics were required. After 30 min of observation, the patient was transferred from the PACU to the ward. The postoperative course was uneventful, and the patient was discharged two days after surgery.

## Discussion

We successfully managed anesthesia in a pediatric patient with trisomy 18 and a potentially difficult intubation orofacial feature using an SGA with preserved spontaneous breathing. The fitting of the SGA was evaluated with a BF. Ventilation was supported by PSV and PEEP. The concomitant IINB was useful for facilitating anesthetic management with spontaneous ventilation by minimizing intravenous opioid administration.

In this case, with a history of intubation difficulties at birth, the SGA was successfully used intraoperatively as the primary airway. SGAs can be effectively used as a primary airway for superficial surgical procedures in children with anticipated difficult intubation or ventilation [3]. In children with trisomy 18, a size-1 laryngeal mask airway (LMA) for a two-month-old boy weighing 3 kg and a size-2 LMA for a three-year-old girl weighing 10 kg were reported to be effective for general anesthesia [5,6]. One review claimed that SGA insertion and subsequent ventilation are usually independent of anatomical factors and other airway techniques such as facemask ventilation and laryngoscope-guided tracheal intubation [8]. On the other hand, a retrospective study reported an association between failed facemask ventilation, failed SGA ventilation, and difficult direct laryngoscope-guided tracheal intubation [9]. In our case, BF evaluation confirmed that the SGA fit well and could be used during general anesthesia, even in a child with 18 trisomy and with potentially difficult airway orofacial features.

Only a few studies have reported intraoperative ventilation management using SGAs in children with difficult airways. In our case, a second-generation SGA was used, and PSV and PEEP were set to facilitate spontaneous breathing. The combined use of PSV and PEEP may improve gaseous exchange and ventilation in patients using SGA; however, they are at risk of gastric insufflation caused by positive pressure [10]. AuraGain is a second-generation SGA that allows the insertion of a gastric tube to prevent gastric insufflation. A randomized crossover study using a second-generation supraglottic device (LMA ProSeal) reported that the combination of PSV and PEEP improved gas exchange and reduced work of breathing in pediatric patients without difficult airways [11]. The combination of PSV and PEEP using a second-generation SGA may be a good choice for managing pediatric patients with difficult airways while preserving spontaneous breathing.

In the present case, IINB was useful for anesthetic management with spontaneous breathing. The efficacy of IINB may also be supported by the fact that pain control was achieved with a relatively low dose of opioids, which decreased the risk of opioid-induced respiratory depression in this case. Right IINB was performed instead of caudal epidural anesthesia because the patient had a sacral cutaneous sinus. Congenital dermatological abnormalities are prevalent in the sacral and caudal regions, with 2%-4% of infants presenting with sacrococcygeal dermal sinuses in the perianal area, called pits or dimples [12]. To the best of our knowledge, trisomy 18 and the sacrococcygeal dermal sinus were not associated. Although a recent meta-analysis revealed that quadratus lumborum and transversus abdominis plane blocks were more effective than other regional analgesia for pediatric inguinal hernia repair [13], ultrasound-guided IINB was also safe and effective [14].

Several cautions must be observed when using SGAs in pediatric patients with difficult airways. First, the SGA should be inserted with sufficient depth of anesthesia to avoid laryngospasm. If laryngospasm occurs, intravenous anesthetics or neuromuscular blocking drugs should be administered to reopen the upper airway. Second, a poor fit after SGA insertion may prevent its use as a primary airway intraoperatively. If ventilation is insufficient after ruling out laryngospasm, the glottis must be checked through the SGA using a BF to ensure it is inserted at the correct position. If the glottis is not visible with the BF after adjusting the SGA position, resizing SGA or discontinuing its use must be considered. Third, patients with significant anatomic abnormalities may have difficulty using SGAs intraoperatively [15]. In these cases, tracheal intubation should be attempted in the best possible manner with a video laryngoscope or BF while maintaining oxygenation. Finally, patients with trisomy 18 may have various comorbidities, such as congenital heart diseases, and their overall condition may vary among cases. In our case, the patient had relatively few complications and was in good general condition. Patients with more comorbidities and more severe illnesses would require more careful management.

## Conclusions

We successfully managed an open repair of the right inguinal hernia in a patient with trisomy 18 using an SGA as a primary airway. The concomitant IINB was helpful for anesthetic management with spontaneous breathing maintained using PSV and PEEP. A second-generation SGA may be a potential alternative as a primary airway for superficial surgery in a pediatric patient with trisomy 18.
